# Exploring Iodide and Hydrogen Sulfide as ROS Scavengers to Delay Acute Rejection in MHC-Defined Vascularized Composite Allografts

**DOI:** 10.3390/antiox13050531

**Published:** 2024-04-26

**Authors:** Philipp Tratnig-Frankl, Alec R. Andrews, Yanis Berkane, Claire Guinier, Marion Goutard, Elise Lupon, Hyshem H. Lancia, Michael L. Morrison, Mark B. Roth, Mark A. Randolph, Curtis L. Cetrulo, Alexandre G. Lellouch

**Affiliations:** 1 Division of Plastic, Reconstructive and Aesthetic Surgery, Vienna General Hospital, Medical University of Vienna, 1090 Vienna, Austria; philipp.tratnig-frankl@meduniwien.ac.at; 2 Vascularized Composite Allotransplantation Laboratory, Center for Transplantation Sciences, Massachusetts General Hospital, Harvard Medical School, Boston, MA 02115, USA; arandrews11@live.com (A.R.A.); claire.guinier@gmail.com (C.G.); marion.goutard0@gmail.com (M.G.); elupon@mgh.harvard.edu (E.L.); hlancia@mgh.harvard.edu (H.H.L.); marandolph@mgh.harvard.edu (M.A.R.); alellouch@mgb.org (A.G.L.); 3 Division of Plastic and Reconstructive Surgery, Massachusetts General Hospital, Harvard Medical School, Boston, MA 02115, USA; 4 Shriners Children’s Boston, Boston, MA 02114, USA; 5 Department of Plastic, Reconstructive and Aesthetic Surgery, CHU de Rennes, University of Rennes, 35000 Rennes, France; 6 Department of Plastic Surgery, NOVO Hospital, 95300 Pontoise, France; 7 Department of Plastic and Reconstructive Surgery, Institut Universitaire Locomoteur et du Sport, Pasteur 2 Hospital, University Côte d’Azur, 06300 Nice, France; 8 Fred Hutchinson Cancer Research Center, Seattle, WA 98109, USA; mmorriso@fredhutch.org (M.L.M.); mroth@fredhutch.org (M.B.R.); 9 INSERM UMRS 1140 Innovation Thérapeutique en Hémostase, University of Paris, 75006 Paris, France

**Keywords:** vascularized composite allotransplantation, transplantation, ischemia–reperfusion injury, acute rejection, free radical scavengers, VCA

## Abstract

Vascularized composite allografts (VCA) face ischemic challenges due to their limited availability. Reperfusion following ischemia triggers oxidative stress and immune reactions, and scavenger molecules could mitigate ischemia–reperfusion injuries and, therefore, immune rejection. We compared two scavengers in a myocutaneous flap VCA model. In total, 18 myocutaneous flap transplants were performed in Major histocompatibility complex (MHC)-defined miniature swine. In the MATCH group (n = 9), donors and recipients had minor antigen mismatch, while the animals were fully mismatched in the MISMATCH group (n = 9). Grafts were pretreated with saline, sodium iodide (NaI), or hydrogen sulfide (H_2_S), stored at 4 °C for 3 h, and then transplanted. Flaps were monitored until clinical rejection without immunosuppression. In the MATCH group, flap survival did not significantly differ between the saline and hydrogen sulfide treatments (*p* = 0.483) but was reduced with the sodium iodide treatment (*p* = 0.007). In the MISMATCH group, survival was similar between the saline and hydrogen sulfide treatments (*p* = 0.483) but decreased with the sodium iodide treatment (*p* = 0.007). Rhabdomyolysis markers showed lower but non-significant levels in the experimental subgroups for both the MATCH and MISMATCH animals. This study provides insightful data for the field of antioxidant-based approaches in VCA and transplantation.

## 1. Introduction

Vascularized composite allografts (VCAs) are a viable reconstruction option for complex tissue defects that cannot be addressed with autologous surgical procedures. To date, more than 200 VCAs have been performed worldwide, making it an emerging field in restoring functionality after extensive injuries [1,2]. The current clinical standard for tissue preservation in solid organ transplantation remains static cold storage (SCS), where donor organs are maintained on ice while preparing the recipient for transplant [2]. Although cold ischemia time is unavoidable during SCS, it is a significant factor in determining the extent of ischemia–reperfusion injuries (IRIs) and graft vasculopathy [3]. In VCAs, cold ischemia can last more than twelve hours, risking graft viability and potentially increasing rejection rates [1].

During ischemia, graft tissues lack oxygen, activating anaerobic pathways primarily in highly metabolic tissues such as muscle-containing VCAs [4]. Previous work demonstrated a 50% loss of viability in porcine latissimus dorsi myocutaneous flaps exposed to nine hours of ischemia [5]. In aerobic conditions, cells physiologically balance the production and elimination of reactive oxygen species (ROS) through homeostatic processes. Following ischemic conditions, reperfusion leads to unprepared oxidative stress, which leads to reactive oxygen species (ROS) [6] production. The release of ROS, along with neutrophil activation, provokes substantial IRI damage in the graft [3]. The implication of IRI in graft rejection has been demonstrated in several models, including VCAs: these reperfusion injuries lead to ischemic cell death and massive antigen release, participating in massive activation of pro-inflammatory pathways and graft allorecognition by innate immunity, eventually leading to rejection phenomena [7,8,9,10]. Multiple preclinical studies have shown that elemental reducing agents such as sulfide, selenide, or iodide, which act as free radical oxygen scavengers in vitro, can improve organ recovery outcomes following oxygen deprivation by decreasing metabolism and IRI [3,4,11,12,13]. These agents also demonstrated promising results in preventing IRI in a swine model of composite tissue autografts [14]. Hydrogen sulfide is a toxic gas at high doses but has endogenous production in mammalian cells with roles in both mitochondrial signaling and ROS scavenging [15,16,17,18].

We hypothesized that treating composite allografts with antioxidants during cold ischemia can mitigate IRI following allotransplantation, decreasing inflammation, tissue injuries, and antigen release. Consequently, this was expected to decrease the immune response, leading to acute rejection. Therefore, the objective of this study was to evaluate the effects of hydrogen sulfide and sodium iodide, as two potential clinically relevant oxygen scavengers, on improving the outcomes, decreasing IRI, and delaying rejection in an MHC-defined swine model of myocutaneous VCA transplantation.

## 2. Materials and Methods

### 2.1. Animal Use and Experimental Design

All experiments were approved by the Massachusetts General Hospital (MGH) institutional animal care and use committee (IACUC protocol 2017N000213) and were in compliance with the National Institutes of Health Guide for the Care and Use of Laboratory Animals and the U.S. Army Animal Care and Use Review Office (ACURO) guidelines. All animals were singly housed in a pathogen-free facility, fed twice daily, and had unrestricted access to water. Major histocompatibility complex (MHC)-defined miniature pigs were used throughout the study [6]. Transplant pairs were assigned based on MHC class I and class II antigen expression, where MATCH group animals (n = 9) were transplanted across minor antigen mismatches (MHC class I and class II antigen-matched), and MISMATCH group animals (n = 9) were fully mismatched for MHC class I and class II antigens. This MHC-defined model was chosen to study the potential impacts of class I and class II antigen mismatch on VCA rejection and assess whether free radical oxygen scavenger effects vary with adaptive immunity in addition to the expected innate response.

**Figure 1:**
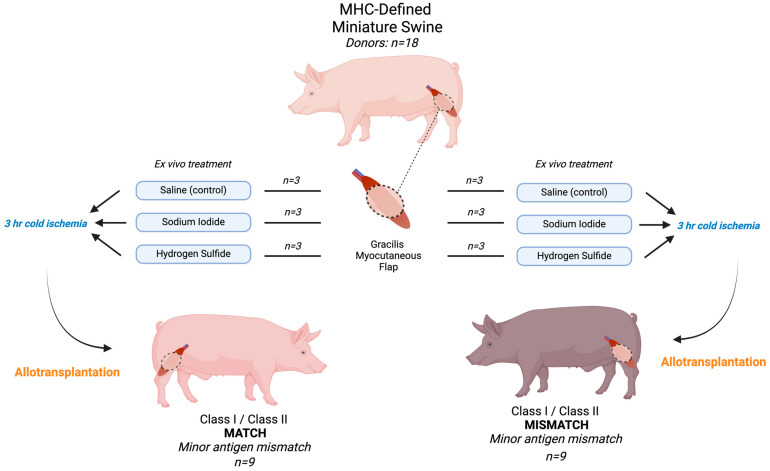
Experimental design and flow chart. All flaps underwent 3 h cold ischemia after receiving the treatment and before allotransplantation. In total, 18 flaps were transplanted. MHC: major histocompatibility complex.

In total, 36 (18 donors, 18 recipients) MHC-defined miniature pigs were used, with an average preoperative weight of 42.8 kg (range: 15–86). Recipients in both MATCH and MISMATCH groups were further divided according to flap treatment, which consisted of either saline (control), sodium iodide (NaI), or hydrogen sulfide (H_2_S) ROS scavenger compound injections. No postoperative immunosuppression was administered to recipients to avoid prolonged flap survival and to study the treatments’ impacts on delaying rejection, and end-of-study time points were determined based on the clinical appearance of the skin paddle and skin biopsies. All flaps were dissected at the end of the study, and skin and muscle samples were formalin-fixed for H&E staining. Histological samples were graded according to the Banff classification for skin-containing VCA [19]. Figure 1 displays the experimental design and study flow chart.

### 2.2. Surgical Model

All surgeries were performed under general anesthesia, following sedation by an intramuscular injection of 2–4 mg/kg of tiletamine hydrochloride and zolazepam hydrochloride and 1–2 mg/kg of xylazine, followed by inhalation of isoflurane (2–3 L/min) for maintaining sedation during the procedure. Our group previously developed and described the myocutaneous flap harvest and transplantation procedures [20]. Briefly, the flap includes the gracilis muscle and an overlying skin island. Flaps were immediately treated according to the assigned group and stored at 4 °C on ice for a total of 3 h cold ischemia while the recipient was prepared for inset. End-to-end anastomoses were performed on the recipient femoral vessels under a surgical microscope. Arterial anastomoses were performed with 8–0 Ethilon (Ethicon Inc., Raritan, NJ, USA) nonabsorbable sutures and venous anastomoses were either performed similarly or with a microvascular anastomotic coupler device (3.5 or 4.0 mm, Coupler, Synovis MCA, Birmingham, AL, USA). Multi-layered wound closure was then performed. A central venous catheter was placed in the external or internal jugular vein of all recipient animals to facilitate postoperative blood collection. Animals were returned to their cage postoperatively and monitored until full recovery. Donor animals could have received on-table transfers to other protocols involving organ harvesting in order to decrease the total number of animals needed within the institution. Donor animals were euthanized at the end of the harvesting procedure following local IACUC and ACURO guidelines.

### 2.3. Flap Treatment

A total ischemia time of three hours began at the restriction of the arterial inflow. After flap procurement, the artery was cannulated with a 22-gauge catheter and flushed with 20 mL of heparinized saline (10 IU/mL), followed by 10 mL of normal saline. Flaps were then treated according to their group assignment. The volume of the treatment compound was calculated using the following equation: Weight of the flap (g) × 0.07 × 5 = Volume of treatment compound flush (ml), based on the approximate blood volume in the flap estimated from the total blood volume. Treatment compounds were administered using a continuous-flow syringe pump set (4100 Pump, Atlanta Biomedical Corporation, Suwanee, GA, USA) with a 6 mL/min flow rate. Afterward, all flaps were stored on ice at 4 °C, as the current gold standard in VCA preservation, until reaching 3 h ischemia time.

### 2.4. Preparation of Oxygen Free Radical Scavengers

Each recipient was assigned to receive either saline control or one of the two experimental compounds, sodium iodide or hydrogen sulfide:

- Saline (control): treatment of the flap with 0.9% saline solution.
- Sodium iodide (NaI): solution containing 10 mg/mL. Animals in this group received an additional intravenous dose of 1 mg/mL one minute after the anastomosis was unclamped.
- Hydrogen sulfide (H_2_S): solution containing 2 mg/kg.

### 2.5. Drinking Water

In addition to the flap treatment, each animal had ad libitum access to drinking water. In the control groups, the drinking water contained 0.9% sodium chloride, whereas in both of the experimental groups, the drinking water contained 280 mmol of sodium iodide to potentialize its scavenging effects, as such impact was demonstrated in earlier work [21].

### 2.6. Blood Analysis

Blood draws for serum isolation were performed daily on postoperative days (PODs) 0–7, 10, and 14 and at the end of the study. Samples were analyzed for blood markers indicating muscle ischemia–reperfusion injury, including aspartate transaminase (AST), creatine kinase (CK), and lactate dehydrogenase (LDH). Each sample was tested using an IDEXX Catalyst One chemistry analyzer (IDEXX Laboratories Inc., Westbrook, MN, USA) according to manufacturer guidelines.

### 2.7. Histopathology

For histological analysis, 6 mm punch biopsies were procured from the donor skin flap on PODs 4 and 10 and at the end of the study. Collected tissues were fixed in 10% phosphate-buffered formalin for at least 24 h. Paraffin blocks were sectioned into 4 µm tick slides for hematoxylin–eosin and digital image capturing. All histology was graded according to the international Banff classification for rejection in skin-containing VCA by an experienced pathologist [19].

### 2.8. Statistical Analysis

Analyses were performed using GraphPad Prism 9.4.0. The significance level was set at 5%. Flap survival means were calculated and compared between groups using Kruskal–Wallis non-parametric tests. Post hoc pairwise comparisons were performed using Dunn’s test. Muscle ischemia marker levels were compared using Student’s *t*-tests.

### 2.9. Endpoints and Recipient Euthanasia

Flap failure was diagnosed on the clinical appearance of the skin paddle (rejection was considered in case of full necrosis of the skin with a deep purple appearance, as defined in previous clinical VCA [22,23,24], and was confirmed by full-thickness histology analysis, following the 2007 Banff classification for skin containing VCA [19]. Recipient animals were euthanized based on a clinical diagnosis of full rejection, following local IACUC and ACURO guidelines.

## 3. Results

In total, 18 myocutaneous flaps were transplanted and evaluated (9 flaps in the MISMATCH group swine and 9 flaps in the MATCH group). One technical failure occurred in the control (saline) subgroup of the MISMATCH group.

**Figure 2:**
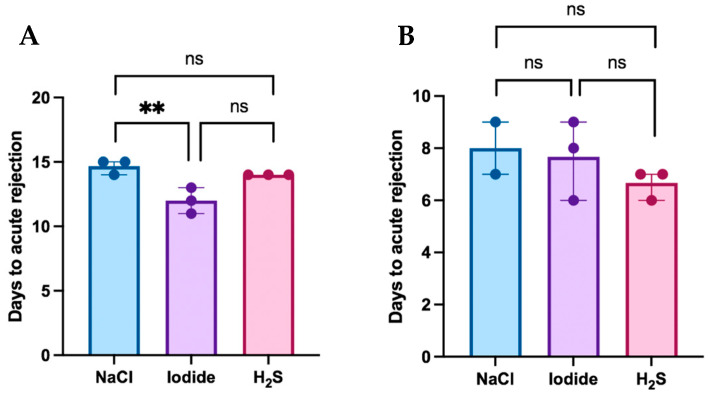
(**A**) Flap survival in the MATCH group. Flap rejection in this group occurred more rapidly in the sodium iodide subgroup when compared to the control (saline). No significant differences were found in flap survival between the hydrogen sulfide subgroup and both the control and iodide subgroups. (**B**) Flap survival in the MISMATCH group. No statistically significant difference was observed in postoperative flap survival according to treatment subgroups in MHC-mismatched animals. **: Statistically significant result; ns: No statistical significance.

Postoperative flap survival in the MATCH and MISMATCH groups is displayed in Figure 2A,B, respectively, according to the treatment compound. In the MATCH group, saline-treated control flaps survived for 15 days postoperatively, while flaps from both treatment groups survived for an average of 13 days (NaI) and 14 days (H_2_S). In this group, flap survival revealed significant differences [H(2) = 6.826, df = 2, *p* = 0.0143]. There was no significant difference in MHC-matched flap rejection between the saline and H_2_S treatments (mean difference = 0.67, *p* = 0.483), but NaI treatment led to complete rejection significantly faster than with saline treatment (mean difference = 5.5, *p* = 0.0296). No difference was found in flap rejection delay between the H_2_S and NaI subgroups. Overall, the MISMATCH animals fully rejected their flaps nine days after inset in the saline and NaI groups, and at seven days in the H_2_S group. There was no significant difference in flap survival when comparing all three treatment groups [H(2) = 1.653, df = 2, *p* = 0.529].

### 3.1. Postoperative Clinical Observations and Pathology Assessment in the MATCH Group

**Figure 3:**
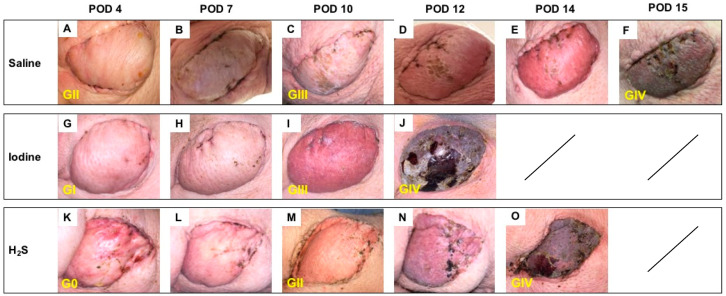
Follow-up in the MATCH group. Representative postoperative appearance of transplanted myocutaneous flaps in MHC class I and class II matched recipient pigs treated with saline (n = 3; **A**–**F**), NaI (n = 3; **G**–**J**), and H_2_S (n = 3; **K**–**O**) on POD 4, 7, 10, 12 and 14 and at the end of the study (**F**,**J**,**O**). The corresponding histological rejection grade (0; I; II; III; IV) based on the 2007 Banff classification is displayed in yellow.

On POD 4, despite minor to no differences in the clinical appearance of the skin paddle, histology showed notable morphological variations between subgroups, with an overall Banff grade II in the control subgroup (Figure 3A) versus an overall grade I in the NaI subgroup (Figure 3G), and no sign of rejection (Banff grade 0) in the H_2_S treatment subgroup (Figure 3K). By POD 10, both controls (Figure 3C) and NaI-treated flaps (Figure 3I) showed Banff grade III rejection, while the H_2_S subgroup was ranked grade II (Figure 3M). On average, the flaps showed a clinical aspect compatible with full rejection, confirmed by grade IV classification, on POD 15 (Figure 3F), POD 13 (Figure 3J), and POD 14 (Figure 3O) in the saline, NaI, and H_2_S subgroups, respectively.

### 3.2. Postoperative Clinical Observations and Pathology Assessment in the MISMATCH Group

**Figure 4:**
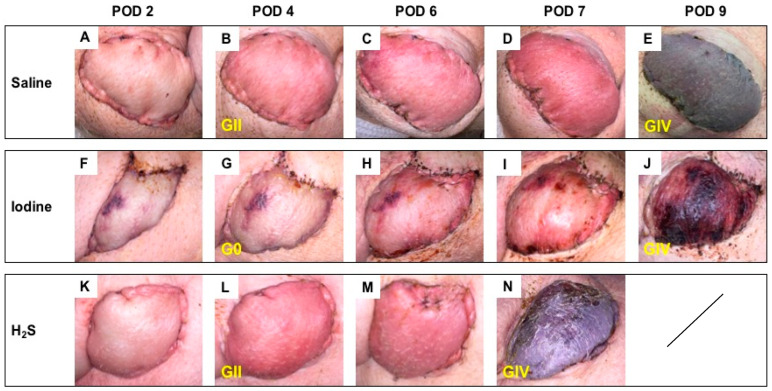
Follow-up in the MISMATCH group. Representative postoperative appearance of transplanted myocutaneous flaps in MHC class I and class II mismatched recipient animals treated with saline (n = 3; **A**–**E**), NaI (n = 3; **F**–**J**), or H_2_S (n = 3; **K**–**N**) on POD 2, 4, and 6 and at the end of the study (**E**,**J**,**N**). The corresponding histological rejection grade (0; I; II; III; IV) based on the 2007 Banff classification is displayed in yellow.

**Figure 5:**
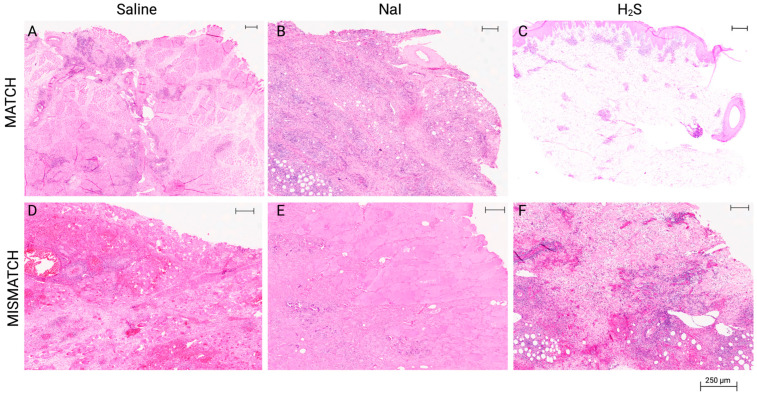
Representative results of histological aspect of the grafts at the end of the study. All final biopsies were procured following clinical diagnosis of full rejection, which was confirmed by the blinded pathology evaluation (Banff grade IV). MATCH group: (**A**) Muscle sample displaying focal infiltration, necrosis, and calcification in the saline subgroup. (**B**) Muscle sample displaying extensive inflammation, endarteritis, and focal muscle necrosis in the NaI subgroup. (**C**) Skin sample displaying grade IV rejection in the H_2_S subgroup (specific 2007 Banff grading: pc3, pa3, ei3, e3, v2, c3, cav0). MISMATCH group: (**D**) Muscle sample displaying frank necrosis of subcutaneous tissue and rhabdomyocytes in the saline subgroup. (**E**) Muscle sample displaying frank necrosis of the skin and subcutaneous tissue in the NaI subgroup. (**F**) Skin sample displaying grade IV rejection (Banff grading: pc3, pa3, ei3, e3, c3, v3, cav0) in the H_2_S subgroup. Hematoxylin and eosin, whole-slide digital imaging, scale: 250 μm indicated.

Clinical appearance on PODs in this group showed signs of rejection as soon as POD 4 (Figure 4). At this time point, the NaI treatment resulted in grade 0 Banff classification (Figure 4G), while both the H_2_S (Figure 4L) and the control subgroups (Figure 4B) were considered grade II. On average, full rejection (Banff grade IV) was observed on POD 9 in the saline and NaI subgroups, while the flaps treated with H_2_S showed full rejection on POD 7. Figure 5 displays representative results of the histological aspect of the grafts at the end of the study (Banff grade IV), and Table 1 provides the detailed scoring as performed by a blinded, experienced pathologist.

### 3.3. Effect of Sodium Iodide (NaI) Treatment on Blood Enzyme Levels in MATCH and MISMATCH Groups

**Figure 6:**
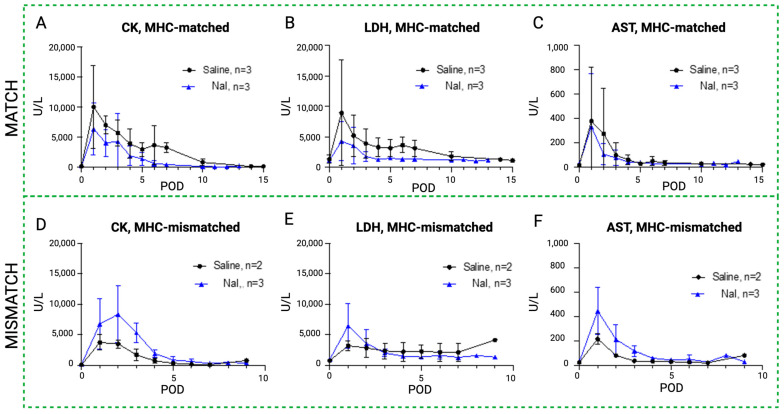
Rhabdomyolysis markers in sodium iodide subgroups versus control. Evolution of systemic serum levels of CK (**A**,**D**), LDH (**B**,**E**), and AST (**C**,**F**) in the NaI treatment group (blue) compared to the control (black) in the MATCH (up) and MISMATCH (low) groups. CK = creatine kinase, LDH = lactate dehydrogenase, AST = aspartate transaminase, POD = postoperative day.

**Figure 7:**
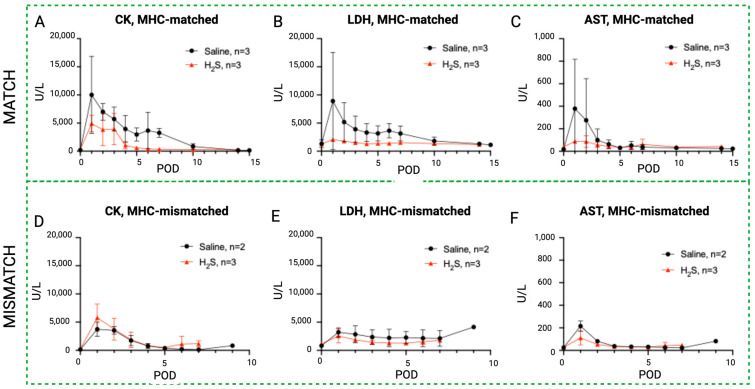
Rhabdomyolysis markers in hydrogen sulfide subgroups versus control. Evolution of systemic serum levels of CK (**A**,**D**), LDH (**B**,**E**), and AST (**C**,**F**) in the H_2_S treatment group (red) compared to the control (black) in the MATCH (up) and MISMATCH (low) groups. CK = creatine kinase, LDH = lactate dehydrogenase, AST = aspartate transaminase, POD = postoperative day.

**Figure 8:**
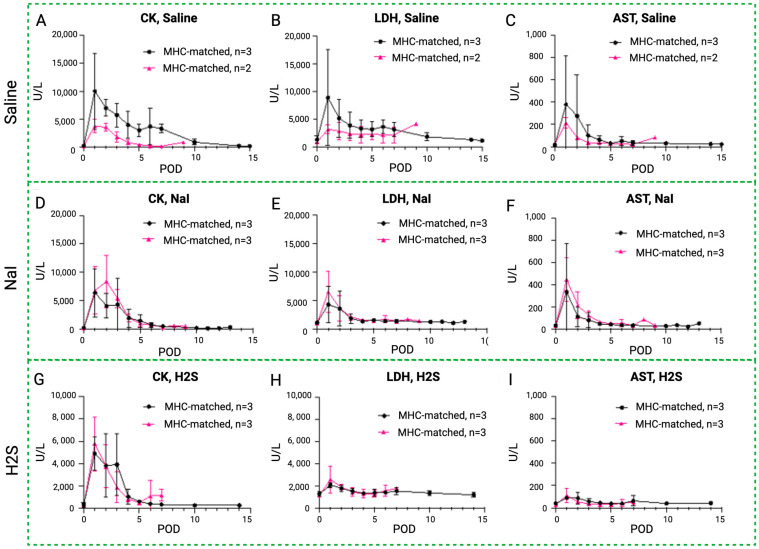
Rhabdomyolysis markers in MATCH and MISMATCH groups. Comparison of CK (**A**,**D**,**G**), LDH (**B**,**E**,**H**), and AST serum level evolution (**C**,**F**,**I**) in MHC-matched (black) and -mismatched (pink) swine according to treatment: saline control (up), NaI (middle), or H_2_S (low). CK = creatine kinase, LDH = lactate dehydrogenase, AST = aspartate transaminase, POD = postoperative day.

The results of the daily serum samples collected from all the study animals are shown in Figure 6, Figure 7 and Figure 8, comparing CK, LDH, and AST as systemic markers of rhabdomyolysis (reflecting muscle ischemia). A decrease in CK (Figure 6A,D), LDH (Figure 6B,E), and AST (Figure 6C,F) levels in both the MATCH (Figure 6A–C) and MISMATCH (Figure 6D–F) groups was found in the control and NaI subgroups. Concentrations peaked early on POD 1–2, followed by a decrease until reaching the baseline level around POD 5. The saline subgroup showed a trend towards higher enzyme levels in the first ten postoperative days compared to the NaI treatment in the MATCH animals. Conversely, in the MISMATCH animals, CK, LDH, and AST levels were higher in the first postoperative days for NaI-treated flaps compared to the saline control recipients. However, none of these differences were statistically significant.

### 3.4. Effect of Hydrogen Sulfide (H_2_S) Treatment on Blood Enzyme Levels in MATCH and MISMATCH Groups

Similar to the NaI treatment subgroup, an initial peak in rhabdomyolysis markers was observed in the H_2_S-treated animals during the early postoperative period before returning to baseline levels (Figure 7). In the MATCH group, the control subgroup again showed a trend toward higher enzyme levels in the first ten postoperative days compared to the H_2_S-treated flaps, but the difference did not reach statistical significance for CK and AST concentrations. On the other hand, LDH levels were significantly higher in saline controls compared to flaps treated with H_2_S. These differences were not found for the MISMATCH recipients, where no notable difference was found between the controls and H_2_S-treated individuals.

### 3.5. Blood Enzyme Levels in MATCH vs. MISMATCH Groups

An initial peak of rhabdomyolysis markers was noted in all groups in the early postoperative period before returning to baseline levels in both the MHC-matched and -mismatched recipients (Figure 8). In the saline control subgroups, CK, LDH, and AST levels were higher in the MISMATCH group compared to the MATCH group, but only the CK levels were statistically significant. In the NaI- and H_2_S-treated subgroups, the CK, LDH, and AST levels followed similar trends, with no significant difference between the MHC-matched and -unmatched animals.

## 4. Discussion

Scavengers for reactive oxygen species have previously been investigated in the setting of oxygen deprivation and ischemia–reperfusion injury. In solid organs, Horvath et al. demonstrated the beneficial effects of methane (CH_4_-) on mitochondrial oxidative capacity in rat livers undergoing cold ischemia followed by 60 min of normothermic reperfusion [25]. Notably, the treated livers showed increased oxygen and glucose consumption, decreased necroenzyme levels, and increased bile production. The authors, therefore, suggested implementing this agent or similar compounds in organ preservation solutions during static cold storage. Since steatotic livers are more sensitive to IRI, Bardallo et al. [26] investigated the effects of PEG35 and glutathione during cold storage and demonstrated increased mitochondrial integrity and ATP production and increased Nrf2 and HO-1 expression, associated with better protection against oxidative stress. In kidneys, oxidative stress plays a major role in activating various pathways, leading to mitochondria degradation [27]. In this context, several antioxidant agents with scavenging properties have been studied to improve graft function following substantial ischemia. Among these, H_2_S demonstrated promising effects in ischemic rodent kidneys by reducing renal dysfunction and pro-inflammatory cytokine release [28,29], as well as by reducing post-ischemia–reperfusion oxidative stress through the preservation of glutathione levels [30]. Multiple studies are aiming at targeting oxidative stress in kidney injury [31].

Focusing on muscle-containing organs, Sodha et al. [32] conducted a study in Yucatan minipig hearts to determine the effects of sodium sulfide as a ROS scavenger in a myocardial IRI model. The authors administered sulfide ten minutes before reperfusing the left anterior descending artery and demonstrated a significant reduction in myocardial infarct sizes. Another group investigated per os sodium iodide in a rodent myocardial infarction model by adding the agent to drinking water 48 h before the myocardial event [21]. This resulted in lower troponin levels and significantly reduced infarct sizes versus the control. These results suggest that ROS scavengers can potentially mitigate the effects of ischemia following reperfusion of muscle tissue. However, preclinical results should be supported by further evidence before considering wide clinical implementation [33]. Similarly, sodium iodide is an elemental reducing agent that was found to reduce heart damage when infused intravenously into mice in a model of acute myocardial infarction [34]. The efficacy of such intravenous administrations at the dose of 1 mg/kg has also been demonstrated in decreasing myocardial ischemia–reperfusion injuries in swine [21].

In the narrow field of VCA, Villamaria et al. [14] performed porcine myocutaneous gracilis flap transplantation following 3 h of cold ischemia. They investigated interim perfusion with H_2_S, which resulted in the diminution of rhabdomyolysis biomarkers (CK, LDH, and AST). We found similar results in our study in the MHC-matched group using the same markers, but with no statistical significance. Comparatively, Fries et al. [35] studied hydrogen sulfide in a musculocutaneous flap model in Swine Leucocyte Antigen (SLA)-mismatched pigs. In their study, H_2_S-treated VCAs were rejected significantly later than controls after postoperative day 6. Their postoperative monitoring included clinical surveillance of the skin paddle by the investigators, searching for erythema, ecchymosis, epidermolysis, or necrosis, which were graded from 0 to 4, as described by Zdichavski et al. [36]. This difference between groups was also confirmed by histology using the same Banff classification, although the microscopic evidence of rejection was not found beyond POD 10. Regarding the NaI treatment, while our results showed a trend toward lower levels of muscle IRI-associated enzymes in the treated flaps, these results were not statistically significant. A U.S. Army-funded study by Wu et al. [37] was registered in 2016 to evaluate ex vivo VCA preservation using both hydrogen sulfide and hydrogen iodide in a non-human primate upper extremity model. Unfortunately, to date, their data remain unreleased, but future communications from their groups could help to greatly improve the knowledge in this specific field.

In this study, we could not demonstrate the effectiveness of H_2_S or NaI treatment compared to the saline controls in delaying acute rejection. Flap survival was not improved by the treatments, and histology results were not significantly different between the groups. Regarding the experimental design, our work was consistent with previous work, based on a similar surgical model, using similar cold ischemia duration and identical ROS scavenger dosing and preparation. Like Fries et al. [35], we did not provide any immunosuppressive regimen since the study was designed to study the potential delaying of acute rejection. We treated 6 flaps with H_2_S (3 in the MHC-mismatched and 3 in the MHC-matched swine), while Villamaria et al. treated 10 flaps with H_2_S. The lack of statistical difference could be due to insufficient power in our study. However, while the number of animals in each subgroup was low (n = 3), we chose a complex animal model based on MHC-defined pigs, allowing us to assess acute rejection in both minor antigen mismatch (MATCH group) and full mismatch configurations. To our knowledge, this is the first study comparing the effects of ROS scavengers depending on major histocompatibility factors in large animals. In addition, within the MATCH group, the H_2_S-treated flaps showed a tendency for faster rejection than the controls (up to 15 days postoperatively in the saline control subgroup versus 14 days in the H_2_S-treated subgroup for MHC-mismatched swine). Here, again, more power could provide insightful data. Additionally, Fries et al. [35] showed an initial clinical–pathological mismatch, with more severe histological Banff rejection signs in the first postoperative days than the clinical flap appearance. This may reflect either a clinical lag with histopathology or a nonuniform progression of rejection throughout the allograft. Similar differences between clinical and pathological grading had previously been reported by Zdichavsky et al. [36], who found that tissues that did not manifest clinical signs of rejection nonetheless showed histopathologic evidence of acute rejection. By implementing both grading systems during the follow-up, we were able to provide a robust rejection assessment in our study. Based on a comparison of our results with previous work, additional preclinical studies are needed to better characterize the effects of hydrogen sulfide and sodium iodide in reducing IRI in VCA. Finally, one major point is the use of a single injection in treatment groups in this study, in contrast with continuous infusion as performed by other authors who showed superiority over a bolus injection in heart models [38,39]. It could be valuable to compare different treatment routes and frequencies in subsequent studies. A particular focus should be given to relevant large animal models [40], recreating the conditions often encountered in clinical VCA settings.

## 5. Conclusions

We evaluated sodium iodide and hydrogen sulfide as two ROS scavengers in the setting of composite tissue allotransplantation in MHC-defined swine. The objective was to determine whether these compounds could mitigate acute rejection by decreasing ischemia–reperfusion injuries. Although obtaining encouraging results in decreasing rhabdomyolysis, potentially due to IRI mitigation, our study was unable to demonstrate substantial efficacy in delaying acute rejection in this model. However, due to the rarity of the model and study design used, these results represent a valuable contribution to the field of antioxidant-based treatments to improve VCA outcomes by selectively testing minor and full major histocompatibility mismatch settings.

## Figures and Tables

**Table 1:** Histological assessment of transplanted flaps at the end of the study. The 2007 Banff grading and component pathological scoring were performed by a blinded, experienced pathologist, with unedited additional comments.

Group	Subgroup	#	EOS Day	Banff Grade	Skin Scoring	Comment (Muscle)
MATCH	Saline	1	15	IV	(pc3, pa3, ei3, e2, v2, c2, cav1)	Focal muscle infiltration, necrosis, and calcification consistent with rejection
		2	15	IV	(pc3, pa3, ei3, e2, v3, c2, cav0)	Extensive perivascular inflammation with endothelialitis
		3	14	IV	(pc3, pa3, ei3, e3, v1, c3, cav3)	Muscle infiltration with focal necrosis
	NaI	4	11	IV	(pc3, pa3, ei3, e3, v3, ct, cav0)	Extensive inflammation, endarteritis, and focal muscle necrosis
		5	12	IV	(pc3, pa3, ei3, e0, v3, c3, cav0)	Extensive inflammation and necrosis
		6	13	IV	(pc3, pa3, ei3, e0, v3, c3, cav0)	Frank necrosis, grade IV with muscle involvement
	H_2_S	7	14	IV	(pc3, pa3, ei3, e3, v2, c3, cav0)	-
		8	14	IV	(pc3, pa3, ei3, e3, v2, c3, cav0)	Grade IV with muscle involvement
		9	14	IV	-	Frank necrosis, grade IV with muscle involvement
MISMATCH	Saline	10	9	IV	(pc3, pa3, ei3, e2, v3, ct, cav0)	Frank necrosis, subcutaneous tissue and muscle
		11	7	III-IV	(pc3, pa3, ei3, e3, v2, c2, cav0)	Diffuse inflammation, focal muscle necrosis
	NaI	13	9	IV	(pc3, pa3, ei3, e2, v3, c2, cav0)	Frank necrosis, skin, subcutaneous tissue and muscle
		14	8	III	(pc3, pa3, ei3, e2, v3, c2, cav0)	Extensive inflammation and necrosis
		15	6	IV	(pc3, pa3, ei3, e3, v2, c3, cav0)	Diffuse necrosis
	H_2_S	16	7	IV	(pc3, pa3, ei3, e3, v3, c3, cav0)	Grade IV with muscle involvement
		17	7	IV	(pc3, pa3, ei3, e3, v3, c3, cav0)	Extensive necrosis with thrombosis
		18	6	IV	(pc3, pa3, ei3, e3, v3, c2, cav0)	Diffuse necrosis

EOS: End of the study day. Pathological component score: (pc) perivascular cells/dermal vessel, (pa) perivascular dermal infiltrate area, (ei) epidermal infiltrate, (e) epidermal apoptosis or necrosis, (v) endarteritis, (c) luminal leukocytes/capillary or venule, (cav) chronic allograft vasculopathy.

## Data Availability

All data can be provided by the authors upon reasonable request.
